# Leveraging Electronic Health Records to Predict the Risk of Acute Kidney Injury after Allogeneic Hematopoietic Cell Transplantation

**DOI:** 10.3390/life14080987

**Published:** 2024-08-08

**Authors:** Elena Bischoff, Nikola Kirilov

**Affiliations:** 1 Faculty of Global Health and Health Care, University “Prof Dr Assen Zlatarov”, 8010 Burgas, Bulgaria; 2 Institute of Medical Informatics, Heidelberg University Hospital, 69120 Heidelberg, Germany; nikola.kirilov@med.uni-heidelberg.de

**Keywords:** electronic health record, allogeneic hematopoietic cell transplantation, acute kidney injury, risk, score

## Abstract

Background: The objective of this study is to assess the electronic health records (EHRs), which are potential risk factors for acute kidney injury (AKI) after allogenic hematopoietic cell transplantation (allo-HCT), and to propose a basic dataset and score for the calculation of HCT-acute kidney injury risk (HCT-AKIR). Methods: We undertook a retrospective analysis of the EHRs of 312 patients. Pre- and post-transplant factors were assessed, recognizing the following structured entries: laboratory data, encounters, medication, imaging studies, diagnoses, and procedures. Composite variables were used to create patient groups by combining two or more multivariate significant risk factors for AKI. The EHRs dataset and HCT-AKIR score were created based on the multivariate analysis of the composite variables. Results: A multivariate analysis showed that previous CKD and once-impaired pre-transplant kidney function, sepsis, imaging procedures with contrast media, and cumulative length of intensive care unit stay after transplantation were significant risk factors. A new unit-weighted composite score based on the combination of significant risk factors contained in common EHR resources was proposed. Conclusions: Using our novel HCT-AKIR score calculated from the basic EHR dataset could be an easy way to increase awareness of post-transplant AKI and provide risk stratification.

## 1. Introduction

Hematologic malignancies are a major public health concern globally and are among the most common types of cancer. [1]. Allogeneic hematopoietic cell transplantation (allo-HCT) is a promising treatment option for patients with certain hematological disorders. The procedure is indicated when all treatment alternatives fail to achieve remission and the reduction of the disease-related risk outweighs the post-transplant morbidity and mortality risk [2,3]. Allo-HCT is a procedure in which stem cells are obtained from a related or unrelated donor who is either human leukocyte antigen (HLA)-matched or mismatched with the recipient. Approximately 50% of allo-HCTs are performed in patients with acute myeloid leukemia, 15% in those with myelodysplastic and myeloproliferative syndromes, and 6% due to primary/secondary bone marrow failure. Less common indications for allo-HCT are lymphoma, myeloma, aplastic anemia, and thalassemia [4]. Nevertheless, the success of the allo-HCT is restricted by a broad spectrum of complications. Sepsis remains a frequent complication in the early pre-engraftment phase after allo-HCT, which often results in multiple organ failure and acute kidney injury (AKI) caused by kidney hypo-perfusion and local intra-renal injuries [2,5,6,7,8]. Furthermore, graft versus host disease (GVHD), infections, and exsiccosis may contribute to the occurrence of AKI, as well as pre-existing conditions and factors, such as comorbidities, age, gender, and nephrotoxic drugs. Recent published studies demonstrated AKI as a common complication after HCT with widely varying incidences due to differences in definitions and populations, suggesting risk factors for the occurrence of AKI after allo-HCT [9,10,11,12,13,14,15]. AKI is a complex disease with clinical manifestations ranging from a slightly elevated serum creatinine level to anuria requiring renal replacement therapy. In 2013, the Kidney Disease Improving Global Outcomes (KDIGO) group published a consensus definition and classification system for AKI combining the Acute Kidney Injury Network (AKIN) criteria for an absolute increase in serum creatinine of ≥0.3 mg/dL within 48 h and the Risk, Injury, Failure, Loss of Kidney Function, End-Stage Kidney Disease (RIFLE) criteria for an increase in creatinine value of ≥50% in a period of 7 days [16,17,18]. The importance of AKI is underlined by its significant role in the development of CKD [19] and the high mortality rate in patients with AKI [20]. The lack of a detailed risk assessment in the previous HCT studies motivated us to identify and propose the basic dataset to calculate the risk of AKI.

Electronic health records (EHRs) are becoming the fuel that propels clinical predictive modeling. Integration of the information contained in EHRs is of utmost importance for the advancement of scientific research [21]. A major challenge for bringing research methods leveraging EHRs into practice is the lack of universal EHRs standards and interoperability problems in healthcare. A step forward is made by the development of the fast healthcare interoperability resources (FHIRs) by Health Level Seven [22]. FHIRs consist of modular components called “resources”, defining a set of data attributes named profiles. Terminologies, such as systematized nomenclature of medicine–clinical terms (SNOMED CT) and logical observation identifiers, names, and codes (LOINC), allow multilingual communication [23].

The objective of this study is to assess the EHRs, which are potential risk factors for AKI after allo-HCT according to the KDIGO definition, and to propose a basic dataset and score for the calculation of hematopoietic cell transplantation-acute kidney injury risk (HCT-AKIR).

## 2. Materials and Methods

### 2.1. Study Design and Definitions

We undertook a retrospective analysis of the EHRs of 312 patients who were hospitalized in the period between January 2012 and December 2014 at the University Hospital of Dresden. Adult patients (18 years of age and older) who received allo-HCT were included in the study. All patient data used were anonymized. The subjects were diagnosed, treated, and followed up until December 2016 by specialists at the Department of Hematology and Stem Cell Transplantation. AKI was defined and staged according to the KDIGO classification as follows: a rise in serum creatinine of more than 1.5 times the baseline value within 7 days. In the absence of baseline serum creatinine the most recent outpatient creatinine value within 90–98 days before baseline was used. If it was missing, the subjects were not included in the study. The three stages included the following: Stage 1: increase in serum creatinine 1.5–1.9× baseline; Stage 2: rise in serum creatinine 2–3× baseline; Stage 3: elevation of serum creatinine > 3× baseline. 

We assessed the following pre-transplant factors: age, comorbidity score, previous CKD, once-impaired kidney function or proteinuria, diabetes mellitus (DM), arterial hypertension (aHT), conditioning regimens, stem cell source, HLA compatibility, and relation to the donor. The comorbidity score was determined on the day of admission according to the “Sorror score” [24]. Among the studied risk factors for AKI after transplantation were: chimerical status on day 14, late chimerical status after one month, engraftment day, sepsis, acute (aGVHD), cytomegalovirus (CMV)-reactivation, immunosuppressive drugs such as cyclosporine A (CsA), tacrolimus, and methotrexate (MTX), imaging procedures with contrast media, and cumulative length of the intensive care unit (ICU) stay.

Systemic Inflammatory Response Syndrome (SIRS) criteria were used to define sepsis. aGVHD was defined as an immune reaction of the donor T cells, which affects the skin, gut, and liver due to the inability of the recipient under myelosuppression to eliminate the immunocompetent T cells of the donor. CMV infection was defined as the detection of CMV-DNA or the expression of the pp65 antigen in blood leukocytes.

### 2.2. Data Collection and Statistical Analysis

To identify risk factors that can potentially predict AKI, we assessed the EHRs generated during the mentioned period. We recognized the following structured data entries: laboratory data, encounters, medication, imaging studies, diagnoses, and procedures. The incidence of AKI was recorded using observations of serum creatinine values at the pre-defined time-points after the HCT procedure. The duration of renal replacement therapy was assessed in cases of AKI requiring dialysis procedures. Encounters were used to identify if patients were admitted to the ICU and to calculate the cumulative duration of the stay at the ICU. Diagnosis of CKD was made before HCT, and repeatedly decreased estimated glomerular filtration rate (eGFR) < 90 mL/min/1.73 m^2^ or increased eGFR > 90 mL/min/1.73 m^2^ with proteinuria over a 3-month period before HCT was extracted. The group of patients with a single measurement of eGFR < 90 mL/min/1.73 m^2^ or eGFR > 90 mL/min/1.73 m^2^ with proteinuria before HCT was defined as “patients with once-impaired kidney function or proteinuria”. Proteinuria was defined as a repeatedly detected urine dipstick test > 0.10 g/L over a period of 3 months. Medication and imaging procedure records were utilized to detect patients who were exposed to contrast agents after HCT. Accordingly, patients were divided into two groups: (1) with at least one imaging procedure with contrast media, and (2) without any imaging procedures with contrast media. In addition, the frequency of imaging procedures with contrast media was counted. Furthermore, the duration of treatment with immunosuppressive drugs (CsA, tacrolimus, or MTX) was calculated. The peak levels in serum of calcineurin inhibitors (CsA and tacrolimus) were extracted using the laboratory data. CsA peak levels greater than 300 ng/mL and tacrolimus peak levels greater than 20 ng/mL were defined as toxic. Two groups were created: (1) with at least one toxic peak plasma level of CsA or tacrolimus, and (2) without toxic peak plasma levels of CsA or tacrolimus. Unstructured data were assessed, and other risk factors were recorded, such as conditioning regimens, stem cell source, HLA compatibility and relation to donor, chimerical status on day 14, late chimerical status after one month, and engraftment day.

Statistical evaluation was performed using SPSS, version 23. The chi-square test and T-test were used to assess the risk factors for AKI. Significant risk factors in the univariate analysis were included in a binary logistic regression (multivariate analysis) to find out whether these risk factors combined have an impact on AKI and if they were independent from other factors. Statistical significance was defined as *p*-value < 0.05. Composite variables were used to create patient groups based on the combination of two or more multivariate significant risk factors for AKI. A basic EHRs dataset and HCT-AKIR score were created based on the multivariate analysis of the composite variables.

## 3. Results

### 3.1. General Subject Characteristics and Incidence of Acute Kidney Injury

The mean age of the subjects was 55.4 years (years) ± 12.98 with a range of 19–78 years. A total of 64.1% (200 of 312 subjects) were male and 35.9% (112 of 312 subjects) were female. The most common diagnosis was acute myeloid leukemia (AML)—160 patients (51.3%). The second most common reason for allo-HCT was myelodysplastic syndrome—48 patients (15.4%). A total of 38 patients (12.2%) underwent transplantation due to Hodgkin’s lymphoma and 25 patients (8%) due to acute lymphatic leukemia. The remaining 13.2% had chronic lymphocytic leukemia, multiple myeloma, osteomyelofibrosis, chronic myeloid leukemia, or other diseases. A total of 116 patients (37.2%) received myeloablative conditioning and 196 patients (62.8%) received reduced conditioning. Of the 312 patients, 163 patients (52.2%) were conditioned with chemotherapy alone. The remaining 149 patients were conditioned with a combination of chemotherapy and total body irradiation (TBI) 2 Gy in 31 patients (9.9%), TBI 8 Gy in 8 patients (2.6%), TBI 12 Gy in 12 patients (3.8%), radioimmunotherapy (RIT) in 5 patients (1.6%), and antithymocyte globulin (ATG) in 93 patients (29.8%). Patient characteristics and pre-transplant factors are presented in Table 1. The type of chemotherapy used before HCT is shown in Table 2; it was not found to have significance for the development of AKI after HCT.

Of the 312 patients, 198 patients (63.5%) developed post-transplant AKI after mean time of 62.5 days. A total of 55 patients (27.8%) were classified as AKI grade 1, 79 patients (39.9%) as AKI grade 2, and 64 patients (32.3%) as AKI grade 3. A total of 122 patients (61.2%) developed AKI only once, 47 patients (23.7%) twice, 15 patients (7.6%)—three times, 8 patients (4.0%)—four times, and 6 patients (3.0%) developed AKI five times after HCT. A total of 312 patients developed AKI 323 times. The mean number of AKI for each patient was 1.6. A total of 131 patients had at least one AKI at the ICU, with a mean duration of stay at 17.3 days. A total of 8 patients (4.04% of 198 patients with AKI) required transient dialysis with an average duration of the stay 20.6 days. Incidence of AKI, stages, and the number of AKIs are shown in Table 3.

### 3.2. Pre-Transplant Risk Factors for Developing AKI

Univariate analysis demonstrated that the major significant factors before transplantation associated with AKI were comorbidity score (*p* = 0.035), aHT (*p* = 0.021), previous CKD, and once-impaired kidney function or proteinuria (*p* < 0.001). The multivariate analysis showed that only previous CKD (*p* = 0.003, odds ratio (OR) 3.244, 95% confidence interval (CI) 1.488–3.224) or once-impaired kidney function/proteinuria (*p* = 0.013, OR 3.635, 95%CI 1.308–10.103), but not the comorbidity score and aHT, were significant factors for post-transplant AKI, demonstrating more than three times increased probability. 79.6% of the patients with CKD (39/49) or 84.4% of the patients with once-impaired kidney function or proteinuria (27/32) before HCT developed post-transplant AKI. A total of 132 (57%) of the 231 patients with normal pre-transplant kidney function developed AKI after HCT. There were no significant differences in gender, age, pre-transplant DM, stem cell source, conditioning regimens, HLA compatibility, or relation to the donor between the two groups of patients—with AKI or without AKI after HCT (Table 4).

### 3.3. Post-Transplant Risk Factors for Developing AKI 

**Figure 1:**
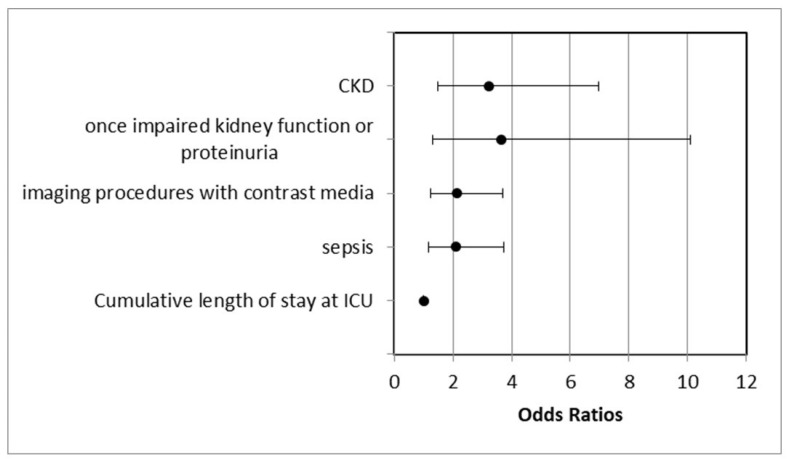
Odds ratios of the multivariate significant factors for AKI: odds ratio (OR) of CKD (3.2), confidence interval (CI) (1.488–6.984); OR of once-impaired kidney function or proteinuria (3.6), CI (1.308–10.103); OR of imaging procedures with contrast media (2.1), CI (1.234–3.690); OR of sepsis 2.1, CI (1.178–3.733); and OR of cumulative length of stay at ICU (1.005), CI (1.000–1.009).

Univariate analysis showed that occurrence of sepsis (*p* = 0.001), performance of at least one imaging procedure with contrast media (*p* = 0.001), at least one toxic CsA or tacrolimus peak plasma level (*p* = 0.013), duration of the therapy with tacrolimus (*p* = 0.009), and cumulative length of the ICU stay (*p* < 0.001) were significantly associated with AKI. Sepsis doubled the risk, and post-transplant AKI occurred in 76.4% (81/106) of the patients. Subjects who underwent imaging procedures with contrast media showed approximately twofold relative likelihood of developing AKI, and it occurred in 76.1% (86/113) of the patients. The mean cumulative length of the ICU stay was significantly longer in the group of patients with AKI (103.4 days) compared to the group of patients without AKI (77.14 days). Every other day at the ICU increased the probability of AKI by 0.5%, and patients who stayed longer than 80 days at the ICU had a twofold increased risk. At least one toxic CsA or tacrolimus peak plasma level was significantly associated with AKI in the univariate analysis (*p* = 0.013), but not in the multivariate analysis, although a certain trend toward significance (*p* = 0.071) has been shown. The duration of the therapy with tacrolimus was not included in the multivariate analysis due to an insufficient number of patients with this kind of data (only 13 patients). There were no significant differences in chimerical status on day 14, late chimerical status, engraftment day, CMV-reactivation, aGVHD, the number of imaging procedures with contrast media, or therapy with MTX and CsA between the groups of patients with AKI and without AKI (Table 5). Pre- and post-transplant risk factors that were significantly associated with AKI in the multivariate analysis and their odds ratios are shown in Figure 1.

### 3.4. Proposal of a Basic EHRs Dataset and Score for the Calculation of HCT-AKIR

**Figure 2:**
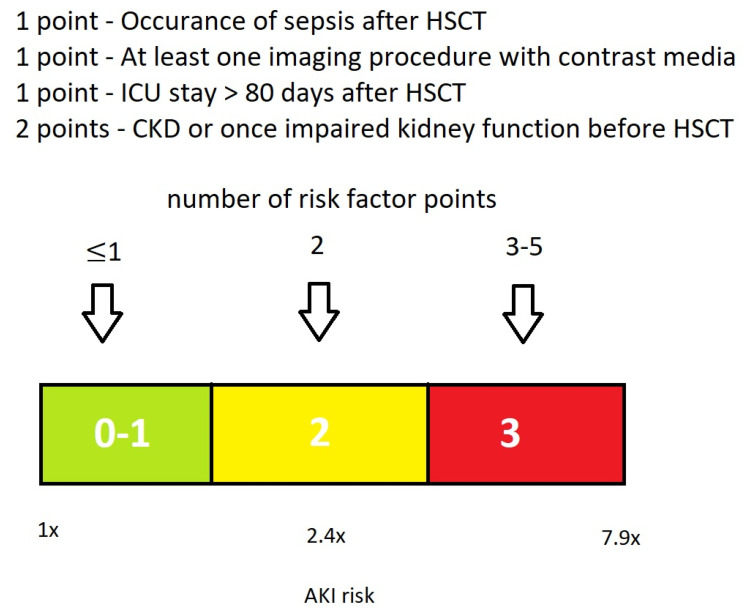
HCT-AKIR score. (green: without risk of AKI, yellow: 2.4-fold risk of AKI, red: 7.9-fold risk of AKI).

All 312 patients were included in the assessment of the HCT-AKIR dataset. The following multivariate significant risk factors for AKI: occurrence of sepsis, performance of at least one imaging procedure with contrast media, and cumulative duration of the ICU stay more than 80 days after HCT were combined to create composite variables of two or more risk factors and were given the same weight (one point), while CKD or once-impaired kidney function before HCT were given double weight (two points) due to their higher predictive power. Patients scoring zero or one point were assigned to composite variable 0, those scoring two points to composite variable 2, and patients with more than three points to composite variable 3. Overall, 66% of the cases were correctly classified (69% correctly predicted cases without AKI and 64.1% correctly predicted cases with AKI). The analysis demonstrated a significant association between the composite variables and AKI. An increase in the odds ratio with an increase in the number of risk factor points was observed. Based on these results, a new HCT-AKIR score can be proposed, ranging from zero to three. Patients with an HCT-AKIR score of 0–1 (without risk factors or one risk factor point) did not show any risk of developing AKI. The probability of developing AKI increases with the HCT-AKIR score: patients with a score of two (two risk factor points) have approximately 2.4-fold increased risk of experiencing AKI, whereas patients with a score of three (more than three risk factor points) have approximately 7.9-fold increased risk of AKI after HCT (Figure 2 and Table 6). The AUC area corresponded to 0.736, with a confidential interval of 0.678–0.793 and a standard false value of 0.029. 

The components of the proposed dataset could be mapped to the available FHIRs resource profiles: Procedure, Condition, Encounter, Medication, MedicationAdministration, DiagnosticReport, Observation and ImagingStudy. Initially, the triggering data point should be an HCT procedure (FHIRs Procedure). The occurrence of sepsis could be detected using patient diagnoses (FHIRs Conditions). Application of contrast media during imaging procedures could be recognized through the patient medication records (FHIRs Medication and MedicationAdministration) and the records in the radiological information system (FHIRs DiagnosticReport and ImagingStudy). An ICU stay and its duration can be identified through recorded encounters (FHIRs Encounter). CKD could be retrieved either by a diagnosis record (FHIRs Condition) or by utilizing the laboratory data (FHIRs DiagnosticReport and Observation) (Table 6).

**Figure 3:**
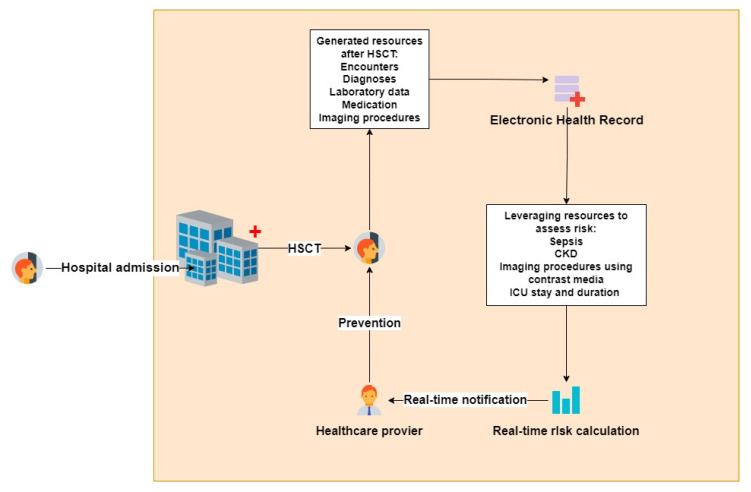
Integration of the HCT-AKIR score in the hospital information technology infrastructure.

The HCT-AKIR can be implemented and integrated into the information technology infrastructure of health care units providing stem cell transplantations. When admitting a patient and performing an allo-HCT procedure, the risk of AKI could be calculated by leveraging the EHRs in real-time. Real-time notifications to healthcare providers could help in the early detection of this severe condition and the prevention of renal replacement therapy (Figure 3).

## 4. Discussion

### 4.1. Incidence of AKI

In our study, we found that two-thirds of all patients experienced AKI after HCT. The incidence of AKI was comparable to that identified by Canet et al. (65%) using the same classification as we did [25]. Only a few studies indicated AKI after HCT according to the KDIGO classification [25,26]. The incidence of AKI reported in previous studies varies substantially due to heterogeneous patient populations with differences in conditioning regimens, classification systems, and follow-up periods used to identify AKI [5,10,11,12,13,20,25,27,28,29,30,31].

### 4.2. Multivariate Significant Risk Factors for AKI

#### 4.2.1. Pre-Transplant CKD or Once-Impaired Kidney Function

Univariate and multivariate analyses showed that pre-transplant CKD and once-impaired kidney function/proteinuria are comparable and independent risks. Approximately 80% of the patients with previous CKD and 84% of the patients with once-impaired kidney function/proteinuria developed AKI after HCT. Therefore, a single abnormal eGFR value preceding HCT, indicating impaired kidney function/proteinuria was equivalently predictive for the occurrence of AKI as CKD. In contrast to our finding, three allo-HCT studies demonstrated that the group of patients with AKI after HCT had a higher eGFR before transplantation compared to the group of patients without AKI [10,30,32]. The reason for these unexpected, counterintuitive results supposed by Kersting et al. may be accounted for by the definition of AKI (more than doubling in serum creatinine); thus, patients with lower creatinine (higher eGFR) before transplantation may have met the criteria for AKI sooner compared to patients with higher creatinine (lower eGFR) [33]. Nevertheless, studies in the general population confirm the association between previous CKD and post-transplant AKI [30,34,35,36].

#### 4.2.2. Post-Transplant Sepsis

The analysis of our study strongly revealed that patients with sepsis have a twofold increased risk of developing AKI. This result is in accordance with most of the HCT studies [25,33,37,38]. However, studies including younger patients (mean age of 18 years ± 13 years with a range of 3 to 59 years and 17 years with a range of 1.5–62 years) [15,39] and pediatric studies [40] compared to our study (mean age of 55.42 years with a range of 19–75 years) did not show any significant association between sepsis and AKI in the univariate analysis. These results and the results of studies in the general population, which demonstrate that the risk of developing post-transplant AKI associated with sepsis increases with age [24,41], indicate that these two complications are dependent on specific patient characteristics.

#### 4.2.3. Post-Transplant ICU Stay and Imaging Procedures with Contrast Media

The cumulative length of the ICU stay was significantly associated with AKI in both univariate and multivariate analyses. Other studies reported admission to the ICU and longer ICU stays as risk factors for AKI in the multivariate analysis as well [11,25], which agrees with our results. 

The performance of at least one imaging procedure with contrast media on a patient after allo-HCT was significantly associated with the incidence of AKI in both univariate and multivariate analyses, and those patients had an independent twofold risk of developing AKI. Exposition to contrast agents in patients after allo-HCT as a risk factor has not been examined in previous studies, and our study is the first one to analyze the contrast media-related risk for post-transplant AKI. Patients with comorbidities are generally at risk of developing AKI and are much more likely to undergo imaging procedures with contrast media; therefore, an accumulation of both risk factors may come into play [42]. A single application of contrast media in patients with multiple other pre- and post-transplant risk factors could determine the occurrence of AKI.

#### 4.2.4. Proposal of a Basic Dataset for the Calculation of HCT-AKIR Score

Despite the various and partially divergent significant risk factors for AKI reported in previous studies, there is no other study that has proposed an EHRs dataset or a risk score model for AKI specifically for patients undergoing an allo-HCT procedure. The predictive risk score suggested by Rodrigues et al. and the new predictive nomogram by Gan et al. were developed for both autologous and allogeneic HCT [43,44]. Moreover, integration of data elements from the EHRs into the scoring system was not discussed. Considering the general importance of AKI for the allo-HCT patient group and the possibility of raising awareness, we believe that the definition of risk factors and their assessment through an easily accessible score could support clinical practice and decision-making. It is a unit-weighted composite score based on the combination of significant risk factors for AKI contained in common EHRs resources.

#### 4.2.5. Weaknesses and Strength of the Study

The major limitation of our study is data collection, which was based on EHRs and unreported conditions could reflect on the accuracy of the results, such as external validation [45].

A major advantage is the accurate and detailed definition of CKD and the long follow-up period of eGFR before HCT. This is the first study, which investigated imaging procedures with contrast media as a risk factor for kidney injury after allo-HCT, and the first one, which indicated a significant association between contrast media administration and AKI. The detailed information about the pre- and post-transplant risk factors for AKI provided by our study allowed us to develop and propose a basic EHRs dataset and HCT-AKIR score.

## 5. Conclusions

This retrospective study of patients undergoing allo-HCT defined an EHRs dataset of specific pre- and post-transplant risk factors for AKI. Patients with once-impaired kidney function/proteinuria at a single time point before transplantation or with preexisting CKD are vulnerable to developing multiple AKIs. Moreover, patients who suffer from sepsis, stay longer than 80 days in intensive care, and undergo at least one imaging procedure with contrast media after HCT are the patient population with the highest risk of developing AKI after transplantation. The novel HCT-AKIR score calculated using the basic EHRs dataset could be an easy way to increase awareness of post-transplant AKI and to provide risk stratification, guiding healthcare providers to avoid serious complications.

## Figures and Tables

**Table 1:** Patient characteristics before allo-HCT.

Characteristics		Patients (N)	Percentage (N = 312)
Gender	male/female	200/112	64.1%/35.9%
Mean age	55.42 years (range 19–78)		
Previous illnesses	aHT	117	37.5%
	DM	51	16.3%
Comorbidity score	0	151	48.4%
	1–2	99	31.7%
	≥3	62	19.9%
Kidney function	Mean eGFR	82.68 ± 12.53 mL/min/1.73 m^2^	
	CKD	49	15.7%
	CKD stage 1	1	0.3%
	CKD stage 2	39	12.5%
	CKD stage 3	9	2.9%
	CKD stage 4 and 5	0	0%
	Ø CKD	263	84.3%
	normal eGFR	231	74%
	once-impaired kidney function or proteinuria	32	10.3%
Hematologic diseases	AML	160	51.3%
	MDS	48	15.4%
	HL	38	12.2%
	ALL	25	8.0%
	CLL	15	4.8%
	other hematologic diseases	26	8.3%
Conditioning	myeloablative	116	37.2%
	reduced intensity	196	62.8%
Conditioning therapy	only chemotherapy	163	52.2%
	ATG	93	29.8%
	TBI 2 Gy	31	9.9%
	TBI 8 Gy	8	2.6%
	TBI 12 Gy	12	3.8%
	RIT	5	1.6%
Stem cell source	PBSCT	271	86.9%
	Bone marrow	41	13.1%
HLA compatibility	10/10 HLA matched	227	72.8%
	9/10 HLA matched	64	20.5%
	haploidentical	21	6.7%
Relation to donor	MSIB	23	7.4%
	MMSIB	2	0.6%
	MUD	204	65.4%
	MMUD	62	19.9%

HT: arterial hypertension; DM: diabetes mellitus; eGFR- estimated glomerular filtration rate by CKD-Epi formula; CKD: chronic kidney disease; AML: acute myeloid leukemia; MDS: myelodysplastic syndrome; HL: Hodgkin lymphoma; ALL: acute lymphoblastic leukemia; CLL: chronic lymphocytic leukemia; ATG: antithymocyte globulin; TBI: total body irradiation; RIT: radioimmunotherapy; PBSCT: peripheral blood stem cell transplantation; MSIB: matched sibling donor; MMSIB: mismatched sibling donor; MUD: matched related donor; MMUD: mismatched unrelated donor.

**Table 2:** Chemotherapeutic agents for conditioning regimens and significance.

Chemotherapy	All Subjects (%)	With AKI (%)	Without AKI (%)	*p*-Value
Cytarabin	201 (64.4)	129 (65.2)	72 (63.2)	0.799
Daunorubicin	126 (40.3)	80 (40.4)	46 (40.4)	0.958
Azacitidin	81 (26)	48 (24.2)	33 (29)	0.338
Mitoxantron	53 (17)	34 (17.2)	19 (16.7)	0.936
Vincristin	52 (16.7)	32 (16.2)	20 (17.5)	0.727
Hydroxydaunorubicin	39 (12.5)	25 (12.7)	19 (16.7)	0.952
Melphalan	35 (11.2)	22 (11.1)	13 (11.4)	0.928
Bendamustin	32 (10.3)	17 (8.6)	15 (13.2)	0.191
Cisplatin	32 (10.3)	21 (10.6)	11 (9.6)	0.808

AKI: acute kidney injury.

**Table 3:** Incidence of AKI, AKI stages, and number of AKIs.

AKI Stages	Patients (N)	Percent of All 312 Patients	Percent of 198 Patients with AKI
Max. Stage 1	55	17.62%	27.8%
Max. Stage 2	79	25.32%	39.9%
Max. Stage 3	64	20.51%	32.3%
Total number of patients with AKI	198	63.5%	100%
Total number of patients without AKI	114	36.5%	-
Number of AKI	Patients (N)	Percent of All 312 Patients	Percent of 198 Patients with AKI
Max. one AKI	122	39.10%	61.62%
Max. two AKI	47	15.06%	23.74%
Max. three AKI	15	4.81%	7.57%
Max. four AKI	8	2.56%	4.04%
Max. five AKI	6	1.92%	3.03%
Total number of patients with AKI	198	63.5%	100%
Total number of AKI	323	-	-

Max.: maximum.

**Table 4:** Pre-transplant risk factors for AKI.

Factor		All Patients (% of 312)	Patients with AKI (% of 198)	Patients without AKI (% of 114)	Univariate Analysis (*p*-Value)	Multivariate Analysis *p*-Value/Odds Ratio (95%CI)
CKD		49 (15.7)	39 (19.7)	10 (8.8)	0.000	0.003/3.224 (1.488–6.984)
Once-impaired kidney function or proteinuria		32 (10.3)	27 (13.6)	5 (4.4)		0.013/3.635 (1.308–10.103)
Normal eGFR		231 (74%)	132 (66.6)	99 (86.8%)		
aHT		117 (37.5)	84 (42.4)	33 (28.9)	0.021	0.072
Comorbidity score		0	151 (48.4)	89 (44.9)	0.035	0.209
		1–2	99 (31.7)	61 (30.8)		
		≥3	62 (19.9)	48 (24.3)		
Age		Mean age (range)			0.074	-
		55.42 years (19–75)	56.47 years (21–75)	53.59 years (19–74)		
Gender	male	200 (64.1)	134 (67.7)	66 (57.9)	0.083	-
	female	112 (35.9)	64 (32.3)	48 (42.1)		
DM		51 (16.3)	37 (18.7)	14 (12.3)	0.141	-
Stem cell source	PBSCT	271 (86.9)	176 (88.9)	95 (83.3)	0.162	-
	Bone marrow	41 (13.1)	22 (11.1)	19 (16.6)		
Conditioning regimens	myeloablative	116 (37.2)	74 (37.4)	42 (36.8)	0.925	-
	reduced	196 (62.8)	124 (62.6)	72 (63.2)		
HLA compatibility	HLA 10/10	227 (72.8)	143 (72.2)	84 (73.7)	0.166	-
	HLA 9/10	64 (20.5)	45 (22.7)	19 (16.7)		
	HLA haploidentical	21 (6.7)	10 (5.1)	11 (9.6)		
Relation to donor	MSIB	23 (7.4)	13 (6.6)	10 (8.8)	0.373	-
	MMSIB	2 (0.6)	0 (0)	2 (1.8)		
	MUD	204 (65.4)	130 (65.7)	74 (64.9)		
	MMUD	62 (19.9)	45 (22.7)	17 (14.9)		

**Table 5:** Post-transplant risk factors for AKI.

Factor	All Patients (% of 312)	Patients with AKI (% of 198)	Patients without AKI (% of 114)	Univariate Analysis (*p*-Value)	Multivariate Analysis *p*-Value/Odds Ratio (95%CI)
Sepsis	106 (34)	81 (40.9)	25 (21.9)	0.001	0.012/2.097 (1.178–3.733)
Imaging procedures with contrast media	113 (36.2)	86 (43.4)	27 (23.7)	0.001	0.007/2.134 (1.234–3.690)
Cumulative length of ICU stay	93.87 days	103.4 days	77.14 days	0.000	0.034/1.005 (1.000–1.009)
“Toxic“ CsA (>300 ng/mL) and tacrolimus peak plasma level (>20 ng/mL)	163 (52.3%)	114 (57.6)	49 (43%)	0.013	0.071
Duration of the therapy with tacrolimus	198.29 days	271.88 days	78.69 days	0.009	Not included
Chimerical status on day 14	80.91%	80.71%	80.23%	0.866	-
Late chimerical status	94.34%	95.39%	92.45%	0.246	-
Engraftment day	20.64 days	20.88 days	20.21 days	0.451	-
CMV-infection	79 (25.3)	53 (26.8)	26 (22.8)	0.464	-
aGVHD	165 (52.9)	107 (54)	58 (50.9)	0.590	-
Number of imaging procedures with contrast media	1.69	1.76	1.48	0.324	-
Therapy with CsA	283 (90.7)	181 (91.4)	102 (89.5)	0.570	-
Duration of the therapy with CsA	272.18 days	257.95 days	298.04 days	0.305	-
Therapy withTacrolimus	43 (13.8)	26 (13.1)	17 (14.9)	0.660	-

CMV: cytomegalovirus; aGVHD: acute graft versus host disease; CsA: Cyclosporine A; ICU: intensive care unit.

**Table 6:** HCT-AKIR EHRs dataset and correspondence of data elements to the FHIRs resource profiles.

Proposed Basic EHRs Dataset		
Record	Content	Corresponding FHIRs
Procedure	allo-HCT	Procedure
Encounter	ICU stay Duration	Encounter
Diagnosis	Sepsis CKD AKI	Condition
Laboratory Data	Proteinuria Creatinine eGFR	DiagnosticReport Observation
Medication	Contrast media	Medication MedicationAdministration
Imaging procedure	Imaging procedure using contrast media	DiagnosticReport ImagingStudy

## Data Availability

The datasets presented in this article are not publicly available due to inclusion of information that could compromise the privacy of research participants.
